# ARIMA model for predicting chronic kidney disease and estimating its economic burden in China

**DOI:** 10.1186/s12889-022-14959-z

**Published:** 2022-12-30

**Authors:** Yining Jian, Di Zhu, Dongnan Zhou, Nana Li, Han Du, Xue Dong, Xuemeng Fu, Dong Tao, Bing Han

**Affiliations:** Department of Biostatistics, School of Public Health, China Medical University, Shenyang, China

**Keywords:** Chronic kidney disease (CKD), ARIMA model, Economic burden, Prediction

## Abstract

**Background:**

Chronic kidney disease (CKD) is an important global public health issue. In China, CKD affects a large number of patients and causes a huge economic burden. This study provided a new way to predict the number of patients with CKD and estimate its economic burden in China based on the autoregressive integrated moving average (ARIMA) model.

**Methods:**

Data of the number of patients with CKD in China from 2000 to 2019 were obtained from the Global Burden of Disease. The ARIMA model was used to fit and predict the number of patients with CKD. The direct and indirect economic burden of CKD were estimated by the bottom-up approach and the human capital approach respectively.

**Results:**

The results of coefficient of determination (0.99), mean absolute percentage error (0.26%), mean absolute error (343,193.8) and root mean squared error (628,230.3) showed that the ARIMA (1,1,1) model fitted well. Akaike information criterion (543.13) and Bayesian information criterion (546.69) indicated the ARIMA (1,1,1) model was reliable when analyzing our data. The result of relative error of prediction (0.23%) also suggested that the model predicted well. The number of patients with CKD in 2020 to 2025 was predicted to be about 153 million, 155 million, 157 million, 160 million, 163 million and 165 million respectively, accounting for more than 10% of the Chinese population. The total economic burden of CKD from 2019 to 2025 was estimated to be $179 billion, $182 billion, $185 billion, $188 billion, $191 billion, $194 billion and $198 billion respectively.

**Conclusion:**

The number of patients with CKD and the economic burden of CKD will continue to rise in China. The number of patients with CKD in China would increase by 2.6 million (1.6%) per year on average from 2020 to 2025. Meanwhile, the total economic burden of CKD in China would increase by an average of $3.1 billion per year. The ARIMA model is applicable to predict the number of patients with CKD. This study provides a new perspective for more comprehensive understanding of the future risk of CKD.

## Background

Chronic kidney disease (CKD) is an abnormal kidney structure or function, which presents for more than three months with specific implications on health [1]. *Kidney Disease: Improving Global Outcomes (KDIGO) Clinical Practice Guidelines* defines CKD as decreased kidney function shown by glomerular filtration rate (GFR) of less than 60 mL/min per 1.73 m^2^, or markers of kidney damage, such as albuminuria or hematuria, lasting for at least 3 months, regardless of underlying cause [2]. In 2017, the global prevalence of CKD was 9.1%, with 697.5 million cases of CKD recorded. Almost one-fifth of patients with CKD lived in China (132.3 million cases) [3]. From 2000 to 2019, the number of patients with CKD in China increased from 98 million to 150 million [4]. CKD also affects the global burden of mortality. In 2017, 1.2 million people died from CKD worldwide. From 1990 to 2017, the global all-age mortality rate of CKD and its complications increased by 41.5% [3]. In China, almost 200,000 people died from CKD and its complications in 2019 [4]. CKD has been recognized as an important global and national public health problem in China.

CKD causes serious economic costs globally. According to the United States Renal Data System, based on the entire United States population, Medicare spending of patients with CKD exceeded $85.4 billion in 2020, which accounted for 23.5% of total Medicare expenditures [5]. According to the China Kidney Disease Network 2016 annual data report, which included almost one million patients with CKD in the analysis, medical expenditure of CKD was $3916 million, representing 6.5% of the overall expenditure of the database [6]. The medical expenditure mentioned above consists the direct economic burden, which mainly includes hospitalization costs, treatment costs and drug costs. In addition, CKD imposes a significant indirect economic burden on patients, which includes lost productivity due to absenteeism, unemployment, disability, and premature deaths [7]. In the United States, the employment rate of dialysis patients between 2008 and 2013 was less than 30, and 38% of patients who were employed in the preceding six months stopped working when dialysis started [8]. At a major Canadian Transplant Centre, the employment rate of kidney transplant recipients decreased from 68.3% before transplantation to 38.3% after transplantation, and the retirement rate increased from 8.3% before transplantation to 18.3% after transplantation [9]. Since CKD places a significant burden around the world, being able to predict the number of patients with CKD in the future and estimate its economic burden is crucial in providing data support to policy makers.

Autoregressive integrated moving average (ARIMA) model is a common time series analysis and prediction model, which calculates the short-term forecast by analyzing the time series of historical data. Time series analysis methods, especially ARIMA model has been widely used in various fields [10–13], such as economics [14] and demography [15], and has also played a great role in medical research. For example, the ARIMA model has been used to predict the epidemiological trends of the Corona Virus Disease 2019 (COVID-19) in 16 countries around the world [16]. It was also selected to forecast the trends of cancer incidence rates in the United States, and the results indicated that the trends for 2015–2020 was downward by 300–550 per 100,000 persons each year [17]. Besides, the ARIMA model was used to predict the incidence of hepatitis B in China, and the results showed that the incidence has seasonal variation and shows a downward trend from 2010 to 2017 [18]. In addition, ARIMA model can be combined with other models and form powerful hybrid time series prediction methods. The MNGM-ARIMA prediction model, which combines the ARIMA model and the Metabolic Nonlinear Grey Model (MNGM), was used to simulate the oil consumption in the United States without COVID-19 pandemic from January 2020 to March 2021 to assess the impact of COVID-19 on the oil consumption in the United States [19].

Common approaches to predict direct and indirect economic burden in the latest literature are the bottom-up approach and the human capital approach. For example, the bottom-up approach and the human capital approach have been used to measure the direct and indirect economic burden of childhood asthma in Portugal [20]. A study from Australia selected the bottom-up approach to calculate the direct economic burden of idiopathic pulmonary fibrosis [21]. A cost-of-illness study has been used the human capital approach to assess the indirect economic burden of diabetes among adults aged 20–79 globally [22].

In this study, the ARIMA model was used to fit the number of patients with CKD from 2000 to 2018, and the number of patients with CKD in 2019 was selected for model test. We used the ARIMA model to predict the number of patients with CKD in China from 2020 to 2025, and estimated the economic burden of CKD based on the predicted number of patients. In addition, the direct economic burden of CKD was estimated by the bottom-up approach, and its indirect economic burden was estimated by the human capital approach. The purpose of this study was to predict the number of patients with CKD and estimate their economic burden in China, which could assess the future risk of CKD in China.

## Methods

### Data

Data on the number of patients with CKD and disability-adjusted life years (DALYs) for CKD in China from 2000 to 2019 were obtained from the Global Burden of Disease (GBD) [4]. The Chinese gross national income (GNI) per capita in 2019 was obtained from the World Bank [23]. The conversion between USD and RMB was based on the exchange rate in 2019 (100 USD = 689.85 RMB) published by National Bureau of Statistics of China [24]. Microsoft Excel 2016 was used to build the database, and R 4.2.0 software was used for statistical analysis.

### ARIMA model

#### Model description

The ARIMA (*p, d, q*) model can be used for predicting future values based on the past values of a variable itself [25]. ARIMA model has three components, including autoregression (AR), moving average (MA), and integration (I). AR stands for autoregressive, which correlates the pattern of the one-time period to its previous time periods. MA stands for moving average, which uses the errors associated with the forecast at a previous time-step to forecast a variable at a later time-step. The integration (I) is the reverse process of differencing to generate the forecast. The ARIMA model contains three parameters (*p, d, q*). Parameter *p* represents the autoregressive term which express the relationship between current and historical values. Parameter *d* represents the number of differencing transformations done to turn the time-series into a stationary one. Parameter *q* represents the moving average term which is used to eliminate random fluctuations. AR (*p*) model represents p-order autoregressive model. MA (*q*) model represents q-order moving average model. The following are the generalized equations of AR (*p*) model (Eq. (1)) and MA (*q*) model (Eq. (2)) [26].

1 $${X}_t={\phi}_1{X}_{t-1}+{\phi}_2{X}_{t-2}+...+{\phi}_p{X}_{t-p}+{\varepsilon}_t$$

2 $${X}_t={\varepsilon}_t-{\theta}_1{\varepsilon}_{t-1}-{\theta}_2{\varepsilon}_{t-2}-\dots -{\theta}_q{\varepsilon}_{t-q}\kern1em$$

where *ϕ*_*i*_ (*i* = 1,2...*p*) is auto-regressive parameter at ith time-stamp, *θ*_*i*_ (*i* = 1,2...*p*) is moving average parameter at ith time-stamp, *ε*_*t*_ is white noise series with zero mean. We can also use the backshift operator to represent AR (*p*) model (Eq. (3)) and MA (*q*) model (Eq. (4)), the equations are as follows [27]:

3 $$\left\{\begin{array}{c}\phi (B){X}_t={\varepsilon}_{t\kern11.75em }\\ {}\phi (B)=1-{\phi}_1B-\dots -{\phi}_p{B}^p\end{array}\right.$$

4 $$\left\{\begin{array}{c}{X}_t=\theta (B){\varepsilon}_{t\kern11.75em }\\ {}\theta (B)=1-{\theta}_1B-\dots -{\uptheta}_q{\uptheta}^q\end{array}\right.$$

where *ϕ*(*B*) is p-order auto-regressive polynomial, *θ*(*B*) is q-order moving average polynomial, and *B* represents the backshift operator，which can be expressed as follows:

5 $${B}^m{X}_t={X}_{t-m}$$

To make non-stationary time series become stationary after differencing transformation is an essential step to prepare the data for use in an ARIMA model. The backshift operator also can be used to represent the process of differencing as follows [27]:

6 $${Y}_t={\left(1-B\right)}^d{X}_t$$

where *X*_*t*_ is a non-stationary time-series and *Y*_*t*_ is a stationary time-series after differencing. Incorporating the Eq. (3), Eq. (4) and Eq. (6) can yield the equation of ARIMA (*p, d, q*) model, which can be expressed as follows [27]:

7 $$\phi (B){\left(1-B\right)}^d{X}_t=\theta (B){\varepsilon}_t$$

#### Modeling process

In the first step, we established a time series using the number of patients with CKD from 2000 to 2018. In the second step, we used the unit root test to check whether this time series was stationary. We chose the Augmented Dickey Fuller (ADF) test to check and to determine the value of the parameter *d*. In the third step, we used the autocorrelation function (ACF) graph and partial autocorrelation function (PACF) graph to determine the value of the parameter *p* and *q*. In the fourth step, we test and diagnose the model by using white noise test of residuals, and we chose the *Box.* test to perform. The model passed the white noise test (*P* > 0.05), indicating that the model is suitable for our selected time series. In the last step, we predicted the number of patients with CKD from 2019 to 2025.

#### Model evaluation

We used the coefficient of determination (*R*^2^), mean absolute percentage error (*MAPE*), mean absolute error (*MAE*), and root mean squared error (*RMSE*) to evaluate the model fitting effects. The relative error of prediction was used to evaluate the prediction effect of the model. The closer *R*^2^ value is to 1, the model fitting effect is better, while other evaluation metrics are lower, the better. Akaike information criterion (*AIC*) and Bayesian information criterion (*BIC*) were used to evaluate the reliability of time series analysis, and the lower the *AIC* and *BIC* values mean that the model is more likely to be considered as a true model.

### Economic burden of CKD

#### Direct economic burden of CKD

Bottom-up approach was used to estimate the direct economic burden of CKD. In this method, the costs came out through the multiplication of unit costs by the quantities used [28]. The following was the method to estimate the direct economic burden [29]:

8 $$\begin{aligned}Direct&\;economic\;burden\;of\;CKD\\ &=\;the\;number\;of\;patients\;with\;CKD\\ &\quad\times annual\;\cos t\;of\;treatment\;per\;capita\;of\;CKD\end{aligned}$$

To estimate the direct economic burden of CKD from 2019 to 2025, we selected the number of patients with CKD in 2019 published by GBD, the number of patients with CKD from 2020 to 2025 predicted using ARIMA model, and the annual cost of treatment per capita of CKD in 2019 published by the National Health Insurance Administration of China.

#### Indirect economic burden of CKD

The indirect economic burden of CKD was estimated using the human capital approach. This approach estimates the indirect economic burden by calculating the potential future productivity losses due to morbidity and mortality [28]. The human capital approach was calculated by multiplying GNI per capita by DALYs of disease and taking productivity weights for different age groups into account [30]. The following was the method to estimate the indirect economic burden of CKD in 2019 [29]:

9 $$\begin{aligned}Indirect&\;economic\;burden\;of\;CKD\;in\;2019\\ &=\;GNI\;per\;capita\;in\;2019\\ &\quad\times\;DALYs\;of\;CKD\;in\;2019\\ &\quad\quad\times\;productivity\;weights\end{aligned}$$

To estimate the indirect economic burden of CKD in 2019, we selected the Chinese GNI per capita in 2019, the DALYs of Chinese CKD for different age groups in 2019 published by GBD, and the productivity weighted for different age groups (0–14 years: 0.15; 15–44 years: 0.75; 45–59 years: 0.80; 60 years or older: 0.10) [30]. In addition, the annual indirect economic burden of CKD from 2020 to 2025 was calculated by multiplying the indirect economic burden of CKD per capita in 2019 by the annual number of patients with CKD from 2020 to 2025 predicted using ARIMA model, the following was the method [29]:

10 $$\begin{aligned}Indirect&\;economic\;burden\;of\;CKD\;in\;2020\;(2021,\;2022,\;...,\;2025)\\ &=\;indirect\;economic\;burden\;of\;CKD\;per\;capita\;in\;2019\\ &\quad\times\;the\;number\;of\;patients\;with\;CKD\;in\;2020\;(2021,\;2022,...,\;2025)\end{aligned}$$

#### Total economic burden of CKD

The total economic burden was calculated by the sum of direct economic burden and indirect economic burden. The following was the method to estimate the total economic burden [29]:

11 $$\begin{aligned}Total&\;economic\;burden\;of\;CKD\\ &=\;direct\;economic\;burden\;of\;CKD\\ &\quad+\;indirect\;economic\;burden\;of\;CKD\\\\\end{aligned}$$

## Results

### ARIMA model

**Fig. 1:**
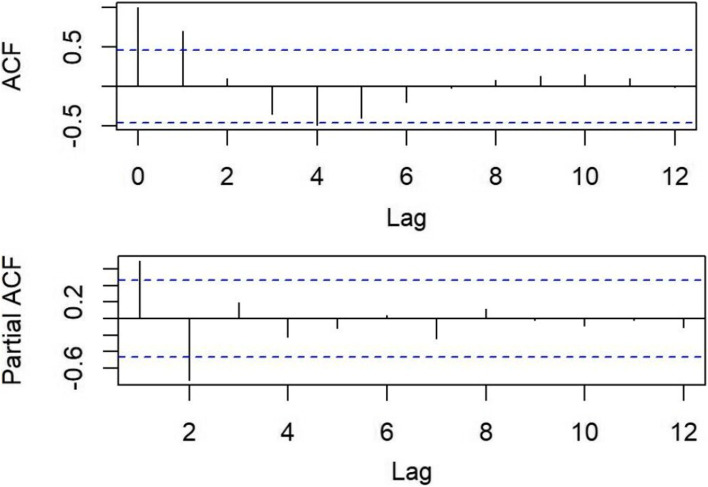
ACF graph and PACF graph of time series after difference transformation

The time series established using the number of CKD patients from 2000 to 2018 showed instability. After the first difference transformation (*d* = 1), the instability of the time series was eliminated and the ADF test showed statistically significant (ADF value = − 3.7018, *P* < 0.05). According to ACF graph and PACF graph (Fig. 1), the autocorrelation coefficient and the partial autocorrelation coefficient were both tailing and were censored after the first order [11], so we obtained the value of the parameters *p* and *q* (*p* = 1，*q* = 1). The ARIMA (1,1,1) model passed the white noise test (*P* > 0.05). The results of *R*^2^ (0.99), *MAPE* (0.26%), *MAE* (343,193.8) and *RMSE* (628,230.3) showed that the model fitted well, and the result of relative error of prediction (0.23%) showed that the model predicted well. *AIC* (543.13) and *BIC* (546.69) also indicated the ARIMA (1,1,1) model is reliable for analyzing this time series. Therefore, we chose to use the ARIMA (1,1,1) model to predict the number of patients with CKD.

### Prediction results

**Fig. 2:**
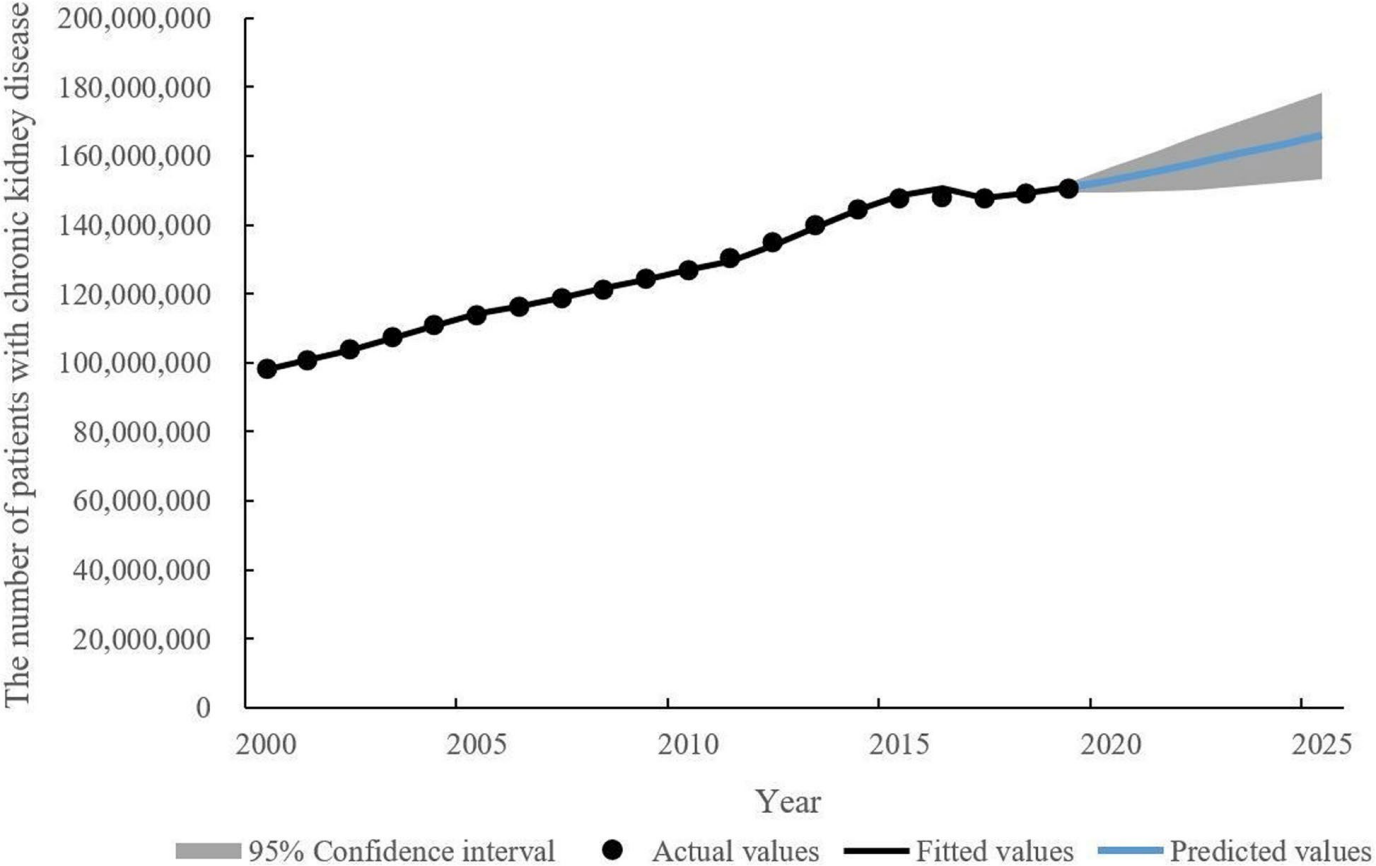
The prevalence trend of CKD in China from 2000 to 2025

We used the ARIMA (1,1,1) model to fit the number of patients with CKD from 2000 to 2018 and to predict the number of patients with CKD from 2019 to 2025 in China, as shown in Table 1. The number of patients with CKD from 2019 to 2025 in China is predicted to be 150,847,832, 153,027,827, 155,428,543, 157,954,788, 160,552,422, 163,190,657 and 165,851,982 respectively. From 2000 to 2025, the number of patients with CKD in China showed a general upward trend, as shown in Fig. 2.

**Table 1:** Actual, fitted, and predicted numbers of patients with CKD in China from 2000 to 2025

Year	Actual values	Fitted/Predicted* values
2000	98,080,324	97,984,936
2001	100,778,482	100,775,432
2002	104,009,758	103,579,484
2003	107,479,258	107,322,311
2004	110,879,984	110,742,468
2005	113,788,685	114,071,395
2006	116,376,516	116,373,592
2007	118,968,752	119,010,713
2008	121,558,481	121,568,753
2009	124,186,392	124,183,625
2010	126,834,134	126,844,106
2011	130,305,497	129,496,697
2012	134,877,033	134,128,886
2013	139,845,490	139,274,367
2014	144,482,052	144,317,596
2015	147,909,807	148,418,959
2016	148,282,232	150,584,633
2017	147,922,520	147,695,698
2018	149,055,942	149,071,942
2019	150,497,490	**150,847,832**
2020	–	**153,027,827**
2021	–	**155,428,543**
2022	–	**157,954,788**
2023	–	**160,552,422**
2024	–	**163,190,657**
2025	–	**165,851,982**

* Predicted values: the numbers in bold are the predicted values

### Economic burden of CKD

We estimated that the total economic burden of CKD in China from 2019 to 2025 was $179,753,462,842, $182,775,685,379, $185,643,089,338, $188,660,423,335, $191,763,025,012, $194,914,119,820 and $198,092,793,471 respectively. The direct economic burden represented a significant portion of the total economic burden of CKD in China, which was $156,312,100,878, $158,940,200,202, $161,433,670,591, $164,057,518,881, $166,755,515,229, $169,495,680,797 and $172,259,828,694 from 2019 to 2025, respectively, as shown in Table 2.

**Table 2:** Direct economic burden of CKD in China from 2000 to 2025

Year	Annual cost of treatment per capita of CKD ($)	Number of patients with CKD ($)	Direct economic burden of CKD ($)
2019	1039	150,497,490	156,312,100,878
2020	1039	153,027,827	158,940,200,202
2021	1039	155,428,543	161,433,670,591
2022	1039	157,954,788	164,057,518,881
2023	1039	160,552,422	166,755,515,229
2024	1039	163,190,657	169,495,680,797
2025	1039	165,851,982	172,259,828,694

According to our estimate, the indirect economic burden of CKD in China in 2019 was $23,441,361,963, and the indirect economic burden per capita was $155.76 in 2019, as shown in Table 3. The estimated indirect economic burden of CKD in China from 2019 to 2025 was $23,441,361,963, $23,835,485,177, $24,209,418,747, $24,602,904,454, $25,007,509,783, $25,418,439,023 and $25,832,964,777 from 2019 to 2025 respectively.

**Table 3:** Indirect economic burden of CKD in China in 2019

Age group	GNI per capita ($)	Productivity weights	DALYs (person-years)	Indirect economic burden of CKD ($)
0–14 years	10,310	0.15	58,398	90,311,889
15–44 years	10,310	0.75	970,617	7,505,299,231
45–59 years	10,310	0.80	1,509,496	12,450,325,489
≥60 years	10,310	0.10	3,293,332	3,395,425,354
Total	–	–	–	23,441,361,963

## Discussion

The results indicate that the number of patients with CKD in China has been increasing since 2000, and reached 150 million in 2019, accounting for more than 10% of the total Chinese population. According to our prediction, the number of patients with CKD in China will continue to increase in the future. The possible reasons for such a large and increasing number of patients with CKD in China are as follows. Firstly, it is related to the etiology and risk factors of CKD. In general, CKD is more common in patients with diabetes and obesity [31]. Diabetes can cause microvascular and macrovascular complications, and microvascular changes within the kidney often lead to CKD [32]. Approximately 35 to 50% of patients with type 2 diabetes will eventually develop kidney damage [33]. China currently has the largest number of patients with diabetes in the world, with an estimated 110 million people affected by diabetes, and type 2 diabetes accounts for more than 95% [34], indicating a large number of patients with CKD caused by diabetes. Furthermore, those who were overweight at age 26 or 36 were about twice as likely to develop CKD compared to general population at the same time [35]. In the past four decades, the rates of overweight and obesity in China increased rapidly [36]. Secondly, the spectrum of CKD in China has been evolving due to rapid economic development and urbanization [37]. In China, the percentage of CKD due to diabetes has exceeded the percentage of CKD due to glomerulonephritis since 2011 [38]. A study based on a large nation-wide dataset in China showed that the proportion of diabetic kidney disease increased from 19.5% in 2010 to 24.3% in 2015, while the proportion of glomerulonephritis decreased from 23.3 to 15.1% [39]. The evolving spectrum of CKD may be related to changing diets and lifestyles. Chronic diseases have become a critical public health problem in China due to rapid urbanization and changes in diet and lifestyle choices [40]. Thirdly, the rapid population aging in China also contributed to the prevalence of CKD. According to the China Kidney Disease Network 2016 Annual Data Report, over one-half of the patients with CKD were 60 years or older [6]. In 2016, there are about 231 million people aged 60 and above in China, accounting for 16.7% of the total population [41]. The number of people aged 60 and above in China rose to 264 million in 2020, accounting for 18.7% of the total population [42]. It is projected that elderly population in China would reach the peak in 2050 [43]. A large elderly population will lead to a higher number of patients with CKD.

In this study, the economic burden of CKD also showed an upward trend from 2019 to 2025. The total economic burden of CKD in China was estimated at about $180 billion in 2019, and according to our prediction, it will increase to $198 billion by 2025. The direct economic burden accounts for more than 80% of the total economic burden of CKD in China. The total economic burden of CKD in 2019 was 1.3% of Gross Domestic Product (GDP) and 18.8% of total health expenditure in China. CKD imposed a heavy economic burden on China for the following possible reasons: Firstly, as a chronic non-communicable disease, CKD has the characteristics of long course, progressive aggravation, difficult to cure and high treatment costs. In China, the median medical expenditure per patient with CKD was $2311 in 2016, which was higher than those without CKD. The average length of stay of inpatients with CKD was also higher than that of patients without CKD, which was 20.33 days per year per patient [6]. Secondly, comorbidities of CKD cause a large economic burden, especially cardiovascular disease (CVD). The prevalence of CVD in patients with CKD is high, and CKD can be considered as a risk factor for development of CVD and increased CVD events [44]. A study in the United States showed that the average total healthcare costs per person per year was higher in CKD patient with comorbid CVD ($37,465) than in those without CVD ($24,271) from 1 January 2007 to 31 March 2019 [45]. Thirdly, compared with the general working population, patients with advanced CKD who received kidney replacement therapies had poorer health and some degree of functional limitation and disability, leading to limited job types, absenteeism and unemployment. A study found that patients receiving kidney replacement therapies were prone to severe fatigue and had poor health status and work capacity. About 30% of employed patients took sick leave for dialysis, and many more drop out of work [46]. Moreover, among kidney transplant recipients who returned to work, their jobs changed from heavy labor to sedentary work, and reliance on government disability insurance increased by 20% [9].

The existing studies on the prevalence trends and economic burden of CKD in China mostly describe and analyze the current situation [47, 48], but lack the prediction of the future situation in China. In 2018, a study predicted the number and prevalence of dialysis patients in China from 2018 to 2025, and the results showed that the prevalence of kidney disease treated with dialysis in China would increase [49]. Consistent with the results of the above study, the results of this study indicated that the number of CKD patients in China would also be on the rise. In addition, our study estimated the economic burden of CKD in China in the coming years. Therefore, this study might provide a more comprehensive assessment of the future risk of CKD in China. Besides, In the United States, a study using the CKD Health Policy Model to predict the prevalence of CKD showed that the prevalence of CKD in adults aged 30 years and above is expected to increase from 14.4% in 2020 to 16.7% in 2030 [50]. In a study from Singapore, the Markov model was used to predict the prevalence and number of residents with CKD, and the results indicated that from 2007 to 2035, the number of residents with CKD was expected to increase from 316,521 to 887,870, and the prevalence would increase from 12.2 to 24.3% [51]. A study developed a dynamic stock and flow model to project the future burden of CKD in Chile showed that both the number of cases and direct economic burden of CKD would increase from 2021 to 2041 [52]. The results of the above studies are consistent with this study, indicating that CKD has become one of the important public health problems worldwide. However, compared with these studies, the ARIMA model has better model accuracy (smaller relative error of prediction) and precision (more specific model effect evaluation indicators) in predicting the number of patients with CKD, and can predict the number of patients with CKD per year and its economic burden in the future. Therefore, ARIMA model can be one of the methods to predict the future prevalence trend and economic burden of CKD.

### Limitations

This study has some limitations. Firstly, in calculating the economic burden of CKD from 2020 to 2025, we chose the treatment cost per capita and GNI per capita in 2019, without adjusting economic factors such as inflation and currency depreciation. Secondly, this study did not use the detailed stages of CKD for analysis. If the data of each stage of CKD can be obtained, the prevalence trend and economic burden of CKD can be analyzed in more detail. These will be our next research direction.

## Conclusion

As a globally recognized important public health problem, CKD already brings a heavy burden to China, and this burden will continue to increase in the future. The ARIMA model is applicable to predict the number of patients with CKD in China. According to our projections, the number of patients with CKD in China will continue to increase in the coming years. The economic burden of CKD also presents an upward trend in China. Therefore, the prevention and control of CKD is facing great challenges in China. To better address these challenges, the following suggestions are proposed in this study: Firstly, regular screening for kidney disease is recommended for high-risk populations for CKD, such as patients with type 2 diabetes, obesity and people aged 60 and older, to achieve early detection, early diagnosis and early treatment, and avoid the progression to end-stage kidney disease or serious complications as much as possible. Secondly, health insurance coverage needs to be expanded to cover the cost of screening for CKD. Thirdly, encouraging people to adopt a healthy lifestyle could decrease the risk of CKD, such as reasonable diet and moderate exercise.

## Data Availability

The data used in this study can be publicly obtained from Global Health Data Exchange (GHDx). GBD Results Tool (2022). https://vizhub.healthdata.org/gbd-results/. The code may be made available from the corresponding author upon request.

## Abbreviations

CKD: Chronic kidney disease
ARIMA model: Autoregressive integrated moving average model
KDIGO: Kidney Disease: Improving Global Outcomes
GFR: Glomerular filtration rate
COVID-19: Corona Virus Disease 2019
MNGM: Metabolic Nonlinear Grey Model
DALYs: Disability-adjusted life years
GBD: Global Burden of Disease
GNI: Gross national income
AR: Autoregression
MA: Moving average
I: Integration
ADF test: Augmented Dickey Fuller test
ACF graph: Autocorrelation function graph
PACF graph: Partial autocorrelation function graph
MAPE: Mean absolute percentage error
MAE: Mean absolute error.
RMSE: Root mean squared error.
AIC: Akaike Information Criterion.
BIC: Bayesian Information Criterion.
GDP: Gross Domestic Product.
CVD: Cardiovascular disease.
KT: Kidney transplantation.
HD: Hemodialysis.
PD: Peritoneal dialysis.
